# Our Summer at Maplewood—Chapter VII.—At Dinner

**Published:** 1875-08

**Authors:** 


### CHAPTER VII.

### AT DINNER.

"I think, cousin Emmeline, there is no dish more usually detestable in America than soup. Were I to describe most truthfully what I have usually found passing under that name, I should call it greasy water well seasoned with pepper and salt, and having in it macaroni, rice, or stringy bits of vegetables,—a mixture both unwholesome and unpalatable. In my cook book I mean to insist that one of the cardinal virtues of good cooking is, that food shall be so prepared as not to taste watery. I have often dined where every article of food upon the table could have been properly labelled "watery," for the soup was watery, the roast was watery, the vegetables were watery, and even the pies and puddings were watery. But this soup which Alice has served to us to-day, would silence the complaints of the most inveterate grumbler. It is lovely to look at, being of a rich color, between amber and ruby, and the flavor is is like a mingling of many good things, whereby something far better has been produced, than is concealed in any single ingredient. You taste neither water, meat, vegetables, herbs or spices. You cannot be sure this, that or the other went to make up the delicious whole. You relish it. It is appetizing, satisfying, and worthy of being called soup. The cook who serves a soup like this is an artist, with fine tastes and keen perceptions; and although neither Alice nor I could give the exact recipe, the general rules for making soup are easily laid down, and each cook must put in the delicate finishing touches according to taste and fancy.

In the first place a soup kettle must be a soup kettle, and nothing else. And unless made with a false bottom it should stand on a rest that raises it from the stove, so that its contents shall not be in danger of burning. Let the cover fit closely. Put into it a beef bone carefully washed and broken, the trimmings of beef steak, veal cutlets, lamb chops, the refuse pieces of cold meat of all sorts, roasted, boiled or fried, also game, fresh fish, the skeletons of roast chicken or turkey, beef steak bones, and all scraps that are good for nothing else,—bearing in mind always that they must be clean and not burned. Never allow the soup kettle to boil; but let it simmer unceasingly. When all the desirable properties are extracted from the bones and meat by this long continued simmering, strain the contents of the kettle into an earthen bowl. Cover, and set aside. In cold weather, soup "stock," as this preparation is called, may be kept for many days, especially if the surface covering of grease is not removed; but in warm weather it should only be kept, and then in a cool place, from one day until the next. Before returning the stock to the soup kettle, carefully remove all the grease, and add barley, rice, macaroni, vermicilli, vegetables and such ingredients as desired, according to the sort of soup to be made. The carrot I consider one of the most important vegetables for soup, as it has a good taste and a rich color, and is wholesome. Tomatoes and onions come next in order, but the latter should be used only for flavoring, and sparingly. The soup which has inspired my observations was, I think, made in this manner: To two quarts of soup stock, six carrots, two tomatoes, one onion, and a pinch of thyme or sweet marjorum were added. When the vegetables were simmered to pieces the soup was strained, the shreds of vegetables thrown away, and the soup returned to the kettle. A table-spoonful of flour stirred to a smooth paste with a little sweet butter, was added when the soup came to a boil, also a little salt and pepper. Care was taken not to overdo the seasoning but to blend everything perfectly. And just before serving, Alice put into the soup-tureen diced toast, that is dry toast, very crisp, cut in small square bits. These bits of toast are best when added to the soup by the hostess just before serving, as then they reach the mouth in a dry, or less soaked condition, and, requiring some mastication, assist the digestion of the soup. Instead of toast, bread may be cut into small bits and fried in sweet butter until brown and crisp, or carrots cut in the same form and stewed until tender, may be added in place of either, or the soup may be served clear. When carrots, tomatoes, and browned bits of meat and bones are not used in preparing soup, a table-spoonful each of flour, butter and sugar browned together, and added to the soup, gives it a rich color and fine flavor. Pure broths of beef, mutton or chicken delicately seasoned with salt and pepper, and having no other ingredients than rice or barley are always good, and in many respects better than more elaborate soups. But broths, as well as soups, should have their distinct flavors, and should not be mere sloppy or greasy gruel, ferociously salted and peppered."

"This leg of lamb, Alice, is done to a turn, which cannot truthfully be said of the average roast. Meat should be put to roast in as hot an oven as it will bear without burning. After the outside is browned and crusted over, imprisoning the juices within, the heat may be somewhat lessened. But I recommend roasting all meats and fowls as quickly as possible. The roast should lie upon rings or rests, which hold it above the drippings in the pan; and water should be kept in the pan solely to prevent the drippings from burning. The practice, so much in vogue, of slopping this water over the roast, to 'baste' it, is very objectionable. It brings out the juices, and toughens and injures the quality of the meat. Salt for the same reasons should not be sprinkled over the meat before, or while roasting. There is one roast, that is veal, which is an exception to the general rule. Being a dry, tasteless meat, I think it is improved by frequent bastings with a well seasoned gravy, to which a piece of butter and a tea-spoonful of sugar have been added. Veal should be cooked more thoroughly than any other kind of meat except pork,—in fact should be cooked to death. Roast veal is best when cold. To boil meats or fowls, put them in boiling water and simmer smartly, until tender. Never allow them to boil fast or furiously, as so doing toughens the meat, and at the same time robs it of its juices. The smallest quantity of water possible should be used in boiling meats, and no salt should be added until the meat is nearly done. The fowl or joint should be covered closely, and frequently turned in the pot while boiling. Ham is best put in cold water and simmered gently until well done. The ham should be placed in the boiler with the skin up, and with sufficient water to cover it. After it begins to boil, fifteen minutes to each pound it weighs is not too much time to give it for thorough boiling. When removed from the water it should be allowed to cool, before it is skinned. After skinning, place it in a pan, cover the outside thickly with white sugar, saturate with vinegar, and brown richly in a moderate oven.

Salted meats, such as corned beef, tongue, etc., if too salt to be boiled in a small quantity of water, should be soaked over night in cold water, which extracts the salt without injuring the meat."

"Kate, in your cook book do you mean to consider the whole bill of fare from beginning to end; or will you select from it here and there, taking up only such articles and subjects as seem to you most needing consideration?"

"To discuss, or even glance at, our whole bill of fare would require more time than I could give were I to live to three score and ten. All I hope to do is to call attention to the importance of the subject, and to suggest such improvements in methods of cooking as have come under my personal observation. I shall not lumber up my cook book with numberless untried recipes, but will endeavor to give the general principles that underlie them all; so that by its aid any woman with an ordinary quantity of brain, will be enabled to prepare all kinds of food in a proper and healthful manner."

"How delicious these onions are, Alice, —tender and sweet, without being overdone into a tasteless mush. I seldom find beets, onions, or cabbage cooked to my liking. Beets are usually underdone, while onions and cabbage are overdone, or served with a supply of water quite unnecessary. Onions, cabbage, turnips and all greens should be gently pressed in a collender until free from water, then placed in a heated dish, and seasoned with salt, pepper and butter. Young dandelions and spinach are delicious so served."

"Emmeline, on how many tables do you find boiled rice, even, which isn't a disgraceful exhibition ot stupid carelessness, or gross ignorance on the part of the cook?

This is one of the several excellent methods of cooking rice: After the rice has been carefully picked and washed add three measures of warm water to one measure of rice, and soak for an hour. Then cook it in the same water, by setting the dish containing it in a strainer and steaming it for one hour. When put to steam add a small quantity of salt, and stir two or three times with a fork during the first fifteen minutes. Rice cooked in this manner will be white and dry, with each grain separate and distinct, yet soft and palatable. If desired, part of the water can be left out, and its equivalent in milk added, when the rice is nearly done cooking. One advantage of this method is, that rice can be boiled in a china bowl, or any vessel preferred.

Ah, here comes rice pudding! As the spoon cuts through the light brown skin a rich, creamy substance appears—not of a pure white, but with a faint tinge of salmon. Each grain of rice is perfect in form and of large size, as if expanded to its utmost capacity; yet so soft that the slightest pressure is sufficient to crush it. This pudding is made of milk, rice and sugar. It contains no raisins, butter or water. The proportions are these: Two quarts of new milk, five ounces of rice, five ounces of sugar. If desired, it may be flavored with lemon, vanilla or nutmeg, and a small pinch of salt should be added. Place it on the range where it will heat slowly. Stir occasionally while the rice is swelling, and when the milk is boiling hot, place the pudding in a moderate oven and bake for one hour, or until the rice is quite soft. Do not stir the pudding after placing it in the oven; but try it to ascertain if the rice is done before removing it. Serve cold. This simple rice pudding, I think, nine persons out of ten would prefer to those messes and mixtures compounded of boiled rice, eggs, fruit and butter, and served warm with sauce. Yet the average cook takes extra pains to make the expensive, indigestible dish, while this simple, wholesome and delicious dish seems to be almost unknown. The error of supposing that nearly every kind of food is improved by the addition of butter seems to be as wide-spread as it is damaging and false. While some dishes are improved for most tastes, by the judicious use of good butter, a vastly greater number are spoiled by the injudicious use of bad butter. And here I wish to say emphatically that I know ot no judicious use to which bad butter can be applied by a housewife, except that of making it into soap. To put bad butter into pastry, puddings and vegetables, does not make the butter good. It simply spoils the pastry, puddings and vegetables. Many dishes are overdone with sweet butter; while others in which it is usually found are much better without it. American cooks have entirely too much faith in the virtue and potency of grease.

Here Emmeline, is a second letter from consin John, or rather a postscript to his former letter, in which he asks me not to forget dry toast. Listen to him :

'In your chapter on bread, etc., I hope you will not neglect the claims of dry toast. I dare say no woman would be willing to confess she did not know how to toast bread ; yet my experience is that good toast is the exception. Bad bread scorched or burned, and sent to the table cold and tough, is as uninviting a dish as can be served, while a plate of crisp, hot toast, properly prepared is more appetizing and tempting to the average man than the daintiest of hot cakes or muffins. Give me a cup of nice coffee or tea, with a plate of crisp, richly browned toast, and firm golden butter for my morning or evening meal, and I will be content with *any* relish you choose to add—even chipped beef; or I will be satisfied with tea and toast alone, if both are at their level best.'

Poor John ! it is too bad that a forlorn old bachelor can't have the small comfort to be found in tea and toast. I cheerfully give him my aid.

To make the meanest toast possible take bread that is a little sour, a trifle raw, or not quite light, and is not fit to eat untoasted. Lay it on a gridiron over a hot fire where it will burn before it even dries. When burned sufficiently to taste bitter spread it with strong butter and pack the slices one above the other, allowing it to become cool before serving, and you have a dish that would ruin the digestion of an ostrich, and that no sensible person should touch. The woman who thinks good toast can be made of bad bread labors under a terrible delusion. Most of the attempts to improve bad bread or bad butter prove futile, as no amount of doctoring can change their normal condition. They generally retain all their original badness in spite of their disguises and conversions. To make the best quality of toast, the bread must be good, but not over light. If stale, it will toast quickly by being held a short distance from a clear fire. If fresh, lay the slices on a grate, or wire frame, in a hot oven until slightly dried. A toasting fork is much better for toasting bread than a gridiron, as, held by a fork, the entire surface of the bread is exposed to the clear fire, and the distance between the toast and the fire can be increased or lessened at will. Serve toast hot in a toast holder, or laid singly on a plate. If the slices are piled one upon another they sweat and become tough and clammy. Most persons prefer to butter toast for themselves, and many dislike it soaked with melted butter."

Alice seemed restless and impatient for the coming of Susannah to open the closed rooms. She expressed this impatience by a quick, nervous tapping of the toe of her kid slipper upon the carpet, and by a frequent pushing back of the stray curls which rested against her cheek. At last she sprang up joyfully, exclaiming : " O, Susannah, what a deal of work you must have had to do this morning. Where shall we go first ? Up stairs, down stairs, or to the lady's chamber ? "

I always do *her* room first, and the drawing-room last, replied Susannah, adding : " Will you come too, Miss Kate ? and perhaps the Madam wouldn't object ? "

Upon this invitation we all followed her up stairs, and through the dimly-lighted passage, to the suite of rooms heretofore mentioned as having been occupied by the late Mrs. Douglass. The main room was large, airy and well-lighted. Opening out of this were two smaller ones—a bedchamber and a dressing-room. The walls were of a soft grey tint, and were hung with paintings, engravings and chromos, The carpet had a grey ground, covered with vines of the trailing arbutus, so perfect that as our feet pressed them, we almost expected the delicious fragrance of the crushed flowers to come stealing up from their mossy bed. Window draperies, easy chairs and lounges were of grey creton, relieved by glowing vines and brightened blossoms. All the furniture was of rosewood, massive, and delicately carved. At the foot of the bed stood a small crib, the lace curtains of which were looped back with rose-colored ribbons. By the side of the crib Alice paused, and, with trembling voice asked, "Did the baby die too?" "O, yes Miss, before the mother. And it was the shock that killed her; I've no manner of doubt," answered Susannah. "You see she was just hanging, sort of even balanced between life and death, when the nurse announced thoughtlessly that the child was dead. At that she gave a little cry, and stretched out her arms, begging for her baby; and from that time she sank rapidly, sobbing and sighing, until she breathed her last. Now, I think if her baby had lived, she wouldn't have died." Susannah paused a moment, wiped her eyes with the corner of her check apron, and continued: "These bureau drawers, Miss Alice, are packed full of the handsomest baby things you ever saw. There are heaps and heaps of gossamer flannels, tiny socks and dainty night-dresses, and other things, all tucked and puffed with lace. Poor, dear lamb, she used to sit there in the sunshine sewing and singing day after day—singing in a low sweet voice a warble of some sort, just fit for a lullaby. And more than once I said to her, 'Miss Helen, I wouldn't make so many of those little things. It isn't a good sign?' Then she would reply, with a blush, 'Why, I think it a very good sign indeed, Susannah. But go away with superstitions. I put no faith in signs and omens. And I don't think she believed a word in any of the signs; but, bless her dear heart, they came true all the same. And I suppose it was all for the best. For it matters little to any of us whether we die to-day or to-morrow or in a few years. The end soon comes anyhow."

"Cousin Kate, what is this?" interrupted Alice, holding up a small, beautifully bound volume, on the back of which, in letters of gold, was the name—"Helen Douglass."

"That," I answered, is a memorial—a tribute to the dead wife, written by the husband during the first weeks of his bereavement. It contains some beautiful things. Wouldn't you like to look it over at your leisure?"

"Indeed, I should," was the reply, and Alice tucked the book under her arm, hugging it closely to her side, as if she regarded it as a rare treasure, while we followed Susannah to the drawing-room.

No sooner were the windows opened, and the room filled with light and sunshine than Alice excitedly exclaimed:

"Mama, look at those pictures—'My Lord' and his mother. Cousin Kate, what does it all mean? Who are these people?"

"That," I answered quietly, "is the portrait of my old schoolmate Jennie Douglass, and this a very good likeness of her son Gerald; while *this* is an idealized but faithful picture of his wife. Wasn't she a beautiful woman?"

"She *is* beautiful," said Alice. "I can't bear to hear you say she *was* beautiful, as if her life was ended. Had I known her, I could never think of her as dead, I am sure."

"Kate," asked Emmeline, "did you suspect that our Switzerland acquaintances were your friends, the Douglasses?"

"I did suspect, in fact was sure of their identity from your very accurate descriptions. Quite a romance, isn't it, Alice? And that *you* should have dreamed of this man the first night you slept under his roof, not knowing of his association with these scenes, seems to me a singular coincidence. But there is no accounting for dreams, or the strange happening of events. The most unlooked for, as well as most unwished for circumstances, sometimes turn our lives into a new channel, and, in after days, we see what we thought at the time a great calamity, was, after all, for our best good."

"Yes," said Emmeline, reflectively, "this life seems a sad muddle. We go groping about in the dark, seeing strange shapes of evil, and stumbling over broken graves, longing for light, and praying for the mists and fogs to roll away; and sometimes catching glorious glimpses of the evil shapes changing to friendly faces, and the broken graves becoming mounds of bloom and greenness. But, Kate, we have been here a whole month, and you have not devoted one hour to my novel. Now, on the first rainy day I shall expect you and Alice to come to my room and spend the entire morning in listening to portions of it. I want your opinion about so many things—even about deciding upon a title for it. To name my first-born and only child, I found an easy matter compared with determining what to call this fledgeling of my imagination. I think a title should be attractive, and at the same time give a hint, at least, of the contents of the book, don't you?"

"Yes, that is well enough in a novel. But in the case of my cook book I have a different opinion. That I intend to slip into libraries and on centre tables as well as into kitchens and have it read by thousands, who would otherwise never look at it, by selecting a title that suggests pleasant light reading rather than a dry dissertation upon cookery. Won't I steal a march upon many an unsuspecting damsel, and teach hundreds how to cook before they realize they are reading a cook book?"

"I never saw a more spiritual face," said Alice, still wrapt in contemplation of the portrait of Helen Douglass. As represented by the artist, she appeared a blonde with delicate features, whose large eyes and full mouth might have had in them a pout of voluptuousness, but for the pure Madonna-like expression of the whole face. A flowing robe of fleecy white added to the effect. Her hands were folded upon her bosom, one of them touching a large cross suspended from her neck by a tiny gold chain. This was the only ornament she wore. Her eyes were raised as if in prayer, and her light wavy hair floated unconfined over neck and shoulders.

"It is indeed, very lovely, and it will be a wicked shame if the second Mrs. Douglass banishes it to the attic," was Emmeline's response to her daughter.

A slight flush appeared on Alice's cheek as she replied, "Mamma, Gerald Douglass looks to me like a man who loving once, would love for ever; but perhaps I can judge better what manner of man he is, after reading this memorial." And Alice left us to peruse in her own room the small gilt-edged volume dedicated to the memory of Helen Douglass.

---

Typhoid Fever.—The most successful treatment of typhoid in Great Britain to-day is said to be to use no medicine whatever. This we have practiced and urged for years. Enteric fever (or typhoid) is essentially a disease of the intestines. It requires very careful nursing and feeding, but in a vast majority of cases, medicine does harm. The best food is pure, sweet, new milk; beef tea, or a raw egg every 12 hours, make a salutary change. A little pure wine and water in very feeble conditions, works well, but can be got along without in 99 cases in 100. If other food disagrees, stick to milk alone; that will stay down and digest if all else fails.

---

Crimes sometimes shock us too much; vices almost always too little.

---

Toil, feel, think, hope. A man is sure to dream enough before he dies without making arrangements for the purpose.

